# Stepping into the role of a doctor: an instrumental case study to explore the experiences of third-year medical students in a simulated general practice clinic

**DOI:** 10.1080/10872981.2025.2538540

**Published:** 2025-07-25

**Authors:** Niki Jakeways, Sharon Markless, Russell Hearn

**Affiliations:** a King’s Undergraduate Medical Education in the Community (KUMEC), Centre for Education, King’s College London, London, UK; b Centre for Education, King’s College London, London, UK

**Keywords:** General practice simulation, role-play, professional identity, junior medical students, uncertainty

## Abstract

Simulation provides an environment in which students can work beyond their competence. Thus even junior medical students can ‘step into the role of a doctor’; to explore and gain more insight into their future role. Role-playing as doctor in a simulated environment has shown to be of value for final-year students, but this has not been studied with third-year students, who may struggle to be active participants in the clinical environment. This research used instrumental case study methodology to explore the educational value of third-year students ‘stepping into’ the doctor-role in a General Practice clinic simulation. Data was gathered via twenty-four interviews with students and tutors, observations and an online student survey. The value of stepping into the role of doctor centered around the experience of responsibility and was associated with feelings of agency; thinking differently with more focus on management and decision-making; insight into, and preparation for, the role of a practitioner, including working with uncertainty. Most students took part in the exercise of adopting the ‘doctor’ role; student buy-in was dependent upon explicit discussion of the challenge, feelings of authenticity and negotiation of concerns. GP tutors facilitating the clinic played a key role in establishing psychological safety and negotiating student concerns. Stepping into the role of doctor is a valuable exercise for junior medical students as it can provide an authentic experience of clinical responsibility that is not available in the clinical setting; and presents an opportunity to deepen student understanding of the practitioner role.

## Introduction

Simulation is a valuable tool within medical education: it allows trainees to practice beyond their competence and take-on more responsibility, in an environment where patient safety is not put at risk [1,2]. This can be of particular benefit for junior medical students who can struggle to be active participants in the clinical setting and to gain experience of clinical responsibility [3]. There is also concern that clinical experience at this stage is often not contextualised through appropriate discussion for optimal learning [3]. Simulation offers debrief time for this as well as the opportunity for students to take on the role of doctor, rather than a medical student. In our experience, junior students can sometimes feel nervous or unsure about whether they should take on the doctor-role in a simulated setting.

During General Practice (GP) simulation clinics for third-year medical students at a large UK medical school, students take turns to consult with simulated-patients, played by actors, under the facilitation of a GP tutor. Each student has the chance to lead a consultation which includes history, examination, considering differential diagnosis and agreeing on a management plan with the patient. Scenarios are varied and range from possible cancer symptoms for which an urgent referral is needed to someone seeking advice after an ankle injury. The full session involves: a 30-minute ‘pre-brief’ with students and tutor which includes icebreakers, a learning needs assessment and; followed by five simulated-patients arriving for appointments every 30 minutes (during which the student ‘sees’ the simulated patient for ~15 mins and then there is feedback and discussion for the remaining ~15 mins); finishing with a 30-minute debrief. If needed students can take a ‘time-out’ and get help from the tutor or their peers. During these sessions, students had asked if they should take on the role and introduce themselves as a ‘doctor’ or if they should remain in their usual role of a ‘medical-student’. This study arose as there is little evidence about whether it is more beneficial for a student to take on the role of a doctor vs that of a medical student in a simulation.

In real-life, students must introduce themselves to patients as a student; however, in a simulation a student may introduce themselves as, and take-on, the role of a doctor. This is an important role-play aspect within simulation. Although simulation has an extensive evidence base for developing both technical and non-technical skills [4–6]; there is a lack of research that explores this aspect of role-play particularly in the earlier stages of training.

Older studies of role-play have shown it can help students develop empathy, interpersonal skills, and understanding of the self [7–9]; however accounts of simulations in the literature do not often specify the role that students adopted. Simulations with final year students, likely to have taken on the role of ‘doctor’, have been shown to help empower students to take clinical responsibility; increase self-confidence, enthusiasm and motivation to learn; aid decision-making and managing uncertainty; and explore interprofessional team-working [10–13]. Thus, role-playing as a doctor might enable an experience of responsibility that has previously been lacking in medical training [10].

Alongside a novel experience of responsibility, stepping into the role of doctor may also be beneficial to help students consider how they are growing into their new role and developing their professional identity as a doctor. Simulated clinical reasoning sessions with third-year students were found to help rehearse and articulate their decision-making pathways and ‘*to practice what it might mean to “think”, “talk” and “perform” like doctors*’ [14](p.251)]. It’s increasingly recognised that there is value in giving attention to how students’ professional identity develops [15,16], and it seems logical that a simulated setting which is student-centred, rather than patient-centred might be the ideal vehicle for this [17]. This issue of whether role-playing as a doctor might act as a vehicle to explore professional identity formation was a key question that this study aimed to explore and acted as a sensitising concept [18].

Although there are potential benefits in terms of greater exposure to feelings of responsibility, and enabling professional identity formation, simulation can also provoke anxiety and discomfort as students perform in front of their peers and worry about making mistakes. In studies where final-year students have taken on the role of doctor, although mostly successful, some students reported a negative experience [12,19]. Therefore, an important consideration in exploring the student experience of role-playing as a doctor is whether this activity is acceptable to students and how it might affect the psychological safety of the simulations.

From the literature it is clear there is much potential for students to understand more about their future role and how to operate in it, by ‘stepping into the role’ of a doctor, but there is no research exploring how junior students respond to this task and whether they are at an appropriate stage for this challenge.

*Thus, this study aimed to explore any value to third-year students ‘stepping into’ the role of a doctor in a simulated setting and enablers of, as well as barriers to, this.*

## Methodology and methods

As each student’s experience of simulation, and their role as a ‘doctor’/student, will be different, this research was set within the interpretive paradigm acknowledging that there are multiple subjective realities which are socially constructed by and between individuals [20,21]. Instrumental case study methodology aims for an ‘intensive, holistic description of the phenomenon’ [22] and a greater understanding of the dynamic processes involved [23] and thus allowed in-depth learning about both how students experienced the phenomenon of stepping into the different role of doctor, and any barriers to, and enablers of making it a positive learning experience.

### Context and participants

At the GP Simulation Clinics, tutors were briefed to explicitly give students the option of acting in the role of doctor or of student. Each cohort participated in three to four Simulation Clinics over the course of the academic year (*n* = 360 in 2019–20 and *n* = 387 in 2020–21). Due to Covid-19, simulations from April 2020 to April 2021 took place online.

### Data collection

To build an in-depth picture that included different perspectives of the experience, data was collected via twenty-one students and three tutor semi-structured interviews, simulation observations and anonymous online student surveys (total 284 responses, average response rate 40%, survey questions listed in Table 1). All interviews were conducted by the deputy course lead (NJ), lasted approximately 30 minutes, and were informed by a topic guide. Simulation observations took place via a video camera in the ceiling and field notes were made.

**Table 1.** Student concerns about role-playing as a doctor anonymous student survey questions.

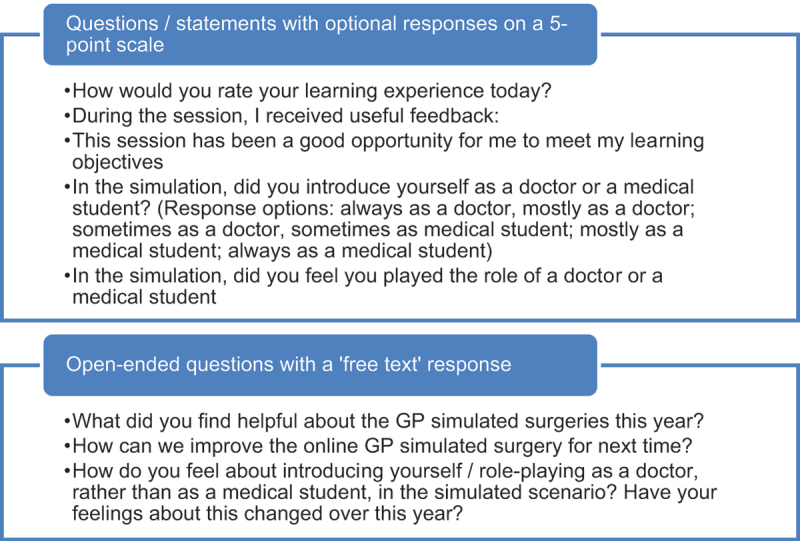	

Semi-structured interviews enabled a deeper and personal exploration of themes and the researcher could pick up on individual cues and elucidate views that were not the social norm [24]. The interview process evolved as data was collected and themes developed, with attention given to the possible effects of a power dynamic where students were discussing aspects of the course with a faculty member. Although some set questions were asked, conversation was valued as an important tool within the dynamic of the interview to allow any unexpected issues to be explored [23].

Ideally a purposive sample of students would have been interviewed [25]; however despite efforts to recruit students with a range of views, most interviewees relayed a positive experience and a response bias was evident. Data saturation was reached in terms of the views of students who had a positive experience of role-playing as a doctor, but less so for negative perspectives. However, the anonymous online survey gave valuable insights into the views of students who did not find the exercise helpful. These used a combination of Likert scale and free-text responses.

The study was approved by KCL Ethics (LRS-18/19–7469), with data was collected with participant consent.

### Strategies to increase trustworthiness

Utilising multiple methods and two different sources facilitated a deeper, more holistic description and analysis of the phenomenon: interviews allowed for deeper exploration of individual student and tutor perspectives; and the anonymity of the online survey allowed more sceptical views to be expressed.

Researcher reflexivity was essential, particularly given the primary interviewer’s faculty position; leading to a potential power dynamic and an ‘insider researcher’ status [26]. A reflexive diary was maintained and fed into the development of the interview process. Open questions at the start of the interview helped to focus it on the participant’s perspective.

### Data analysis

Qualitative data was recorded, transcribed, anonymised, organised using NVIVO, and analysed into themes using Braun and Clarke’s [27] process incorporating techniques such as concept maps, writing, and discussion. Coding was independently reviewed by two faculty peers. This and the process of ‘member checking’ with participants [28] built in further rigour.

## Results

**Figure 1.**
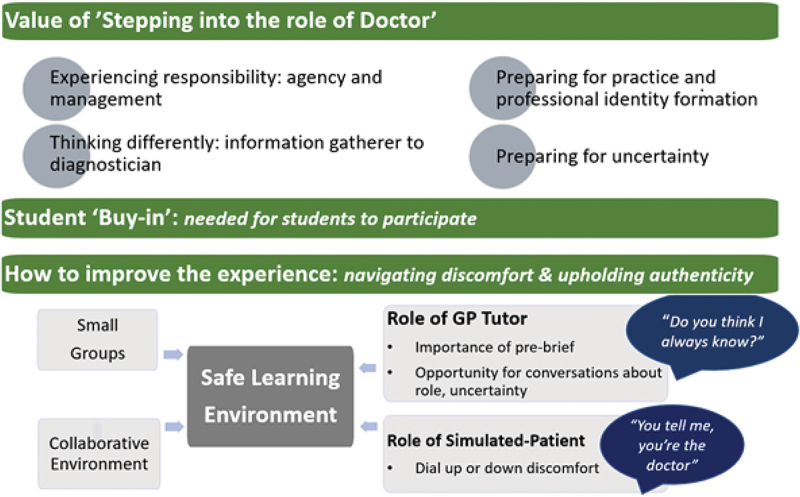
Experience of ‘stepping into the role of doctor’ – summary of results.

These results are organised to first describe the context and the proportion of students who chose to role-play as doctors, followed by an exploration of the value of this experience; and the factors influencing student ‘buy-in’ for the exercise. These results are summarised in Figure 1.

### Context & proportion of students that acted in the doctor role

The simulations were well-received with > 90% students rating the learning experience for all sessions good/excellent via the online questionnaire.

Of the 21 students interviewed, 20 (95%) reported acting in a ‘doctor’ role in the simulation; and 59% students responding to the anonymous online survey felt they ‘always’ or ‘mostly’ role-played as a doctor.

It was clear from the observations, survey and interviews that role fluidity was common, with students appearing to and reporting that they were transitioning between student/doctor roles. Some students commented that they weren’t focussed on their role, but more on being themselves, the dynamic with the patient and what they were doing.

### Value of the experience of ‘stepping into the role of doctor’

Students, tutors and observations identified different aspects of adopting the role of a doctor as valuable and these were categorised into four themes.

#### Experiencing responsibility: agency and management

Nearly all students interviewed felt an enhanced level of responsibility and felt that acting as a doctor allowed them to perform tasks such as discussing management plans with more authenticity. There was a sense of adopting a more active, rather than a passive, role: *‘we always joke about how we feel useless most of the time … it’s quite exciting when you actually do something that’s helpful’* (Student Interview (SI)-19)

Many commented on how the responsibility made them more aware of the importance of safety netting, highlighted knowledge gaps and motivated them to improve: *‘I have the responsibility to make sure that you get the care you need … that falls to me and I’ve got to safety net all around that’* (SI-2)

#### Thinking differently: from information gatherer to diagnostician

Tutors observed and students noted that stepping into the doctor-role prompted them to ‘think harder’ with more focus on potential diagnoses and greater awareness of the consequences of their actions.

*the increased consequences … if you pretended you were in the situation, you really were trying to make sure you’d asked every question you could think of, you were really checking your differentials, … I was thinking harder than I might have done if it was just a, ‘Oh, I’m going to talk to you before you go to the doctor’, which is our normal scenario* (SI-12)

Some students noted that it was hard to attribute this shift solely to the simulation and that it also aligned with progression on the course; however others felt the change in thinking prompted by the simulation was then applied in real clinical situations: ‘*I’ve started . imagining what would I do, … if I was the GP … what would I do in a few years time with the knowledge that I have*’ (SI-17)

#### Preparing for practice and professional identity formation

Students recognised the value of the experience as preparation for their future role; both to help focus on what the role will involve and to build confidence through practice: ‘*it helps us to think about that as the end goal and to focus on that as the target”* (SI-5)

Students interviewed were introduced to the idea of professional identity (PI) formation and some subsequently reported feeling that the experience of acting in a doctor role helped them to think about their own developing PI. *‘today was … helpful for … growing into the identity of a doctor’*(SI-16)

Observational data showed evidence of discussion of many themes pertinent to PI formation including challenge of the paternalistic model; the doctor-patient relationship; holistic care; a doctor’s role and the nature of a ‘good’ doctor.

#### Preparing for uncertainty

Both interviews and surveys demonstrated that a common concern of students was that they lacked the necessary competence and knowledge to role-play as a doctor. Conversations with tutors exploring this could lead to helpful discussion about strategies to manage this: *‘we’re giving them a bit more of that feeling of being responsible and not knowing’* (Tutor (T)-3).

Tutors role-modelled how they dealt with uncertainty and might identify their own learning needs. This opened up an opportunity to challenge assumptions of certainty in their future professional role, and start important discussions about uncertainty:

*they said, ‘Oh I didn’t know what to do there,’ and I said, ‘Well do you think I always know what to do either,’ and then they’d be like, ‘Oh no’ … it opens up a nice conversation (T-3)*

#### Student buy-in: authenticity and discomfort

Although many students found adopting the doctor-role acceptable and associated it with increased confidence and motivation, some reported discomfort. In the online survey 58% respondents described educational value from the exercise of role-playing as a doctor; however 22% reported too much discomfort and not wanting to role-play as a doctor and preferred to role-play as a student. Student concerns were relayed in both the survey and interviews; and centred around the need to maintain authenticity of self; and feelings that students weren’t yet prepared with the necessary skills and knowledge. These concerns are detailed further in Table 2.

**Table 2.** Student concerns about role-playing as a doctor.

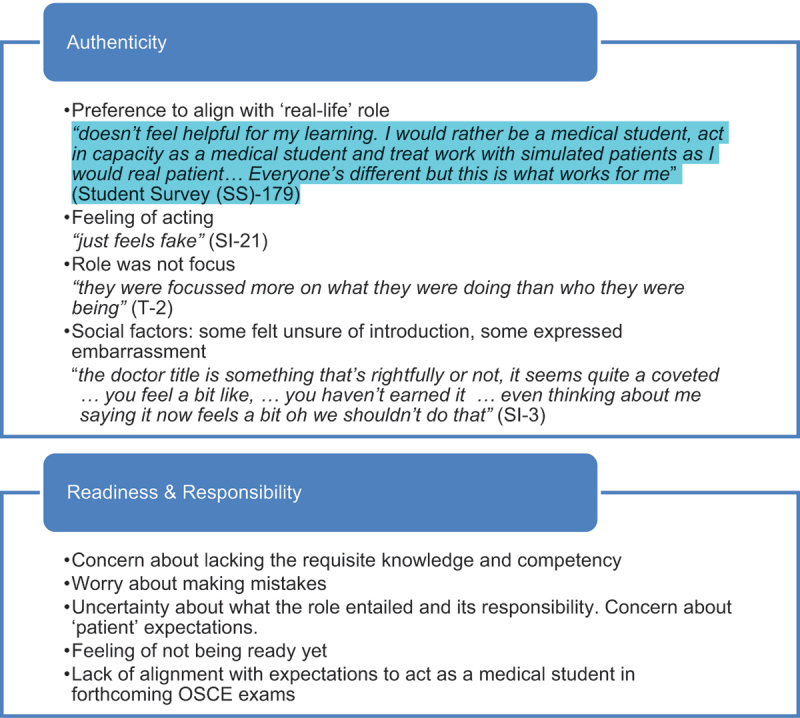	

Discomfort wasn’t always seen as a negative, and 15% survey respondents recognised that this feeling of discomfort might be a useful experience and a sign that they might be learning: ‘ *… worry … I didn’t try and decrease it, because I thought that would be useful learning opportunity, to be quite worried about seeing a patient and having to make a decision’*(SI-12)

#### Student buy-in: navigating concerns & practice pointers

Student buy-in was centred around **facilitation by the GP Tutor within a safe learning environment**. Students cited the value of **small groups, collaborative atmospheres** and consistency of group participants and tutor: ‘*it’s a role-play … everyone’s there to support you, you’re here to learn so even if you … introduce yourself as a doctor and you make mistakes it’s fine … everyone makes mistakes, we’re here to learn”* (SI-6)

Students often recognised that their tutor’s positive facilitation helped them feel comfortable to take-on the new role. It was noted from tutors, students and observations that many tutors recognised students’ reticence and then **pro-actively encouraged**: ‘*I haven’t had any qualms about asking them to do it … sometimes they look a bit shocked … but then when you explain again they’re quite onboard to try and give it a go*’(*T*-3)

**Tutors played a key role in identifying and negotiating student concerns around lack of knowledge, how to introduce themselves, and learning from mistakes**. The simulated patient also played an important role with six students describing how an authentic ‘patient’ response improved fidelity and the challenge of the experience. How common student concerns were negotiated is summarised in Table 3 and practical pointers for tutors are summarised in Table 4.

**Table 3.** How common student concerns were negotiated.

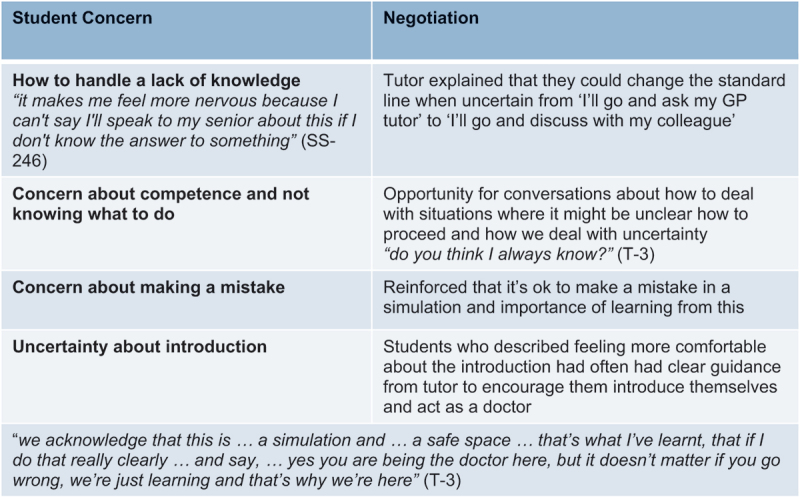

**Table 4.** Practical pointers for tutors to encourage students to ‘step into role of doctor’.

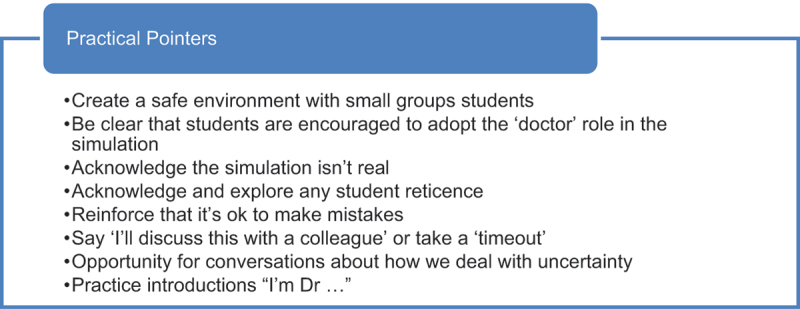	

## Discussion

This research has found that third-year medical students find the opportunity to adopt the role of doctor in a simulated setting both acceptable task and educationally valuable, but they often need encouragement to do so. Attention to student buy-in is key and involves navigating authenticity and discomfort to create a safe learning environment.

### A novel opportunity to experience responsibility and align learning with future career

When taking on the doctor-role students experienced an enhanced level of responsibility transitioning from ‘information-gatherer’ to a more active role using clinical reasoning and synthesising management plans. This was often a novel experience of clinical responsibility and underlines the value of simulation in enabling an experience that does not frequently or safely occur in real clinical settings. This study demonstrated this valuable experience is accessible to junior students earlier in their training than previously thought [3]. Clinical reasoning is a key medical skill, yet how students might optimally learn this is debated [29]: promotion of a more active role could be a useful tool to promote students’ use of clinical reasoning.

Students often reported positive emotions such as increased confidence and motivation; and reinforcing their focus on the overarching goals of medical education. Tapping into these goals is a powerful educational tool: this exercise has demonstrated a means of improving constructive alignment of the curriculum enabling students to focus on their desired outcomes [30].

### Navigating authenticity and psychological safety

Creating a psychologically safe environment is critical as students navigate authenticity and discomfort to act in the doctor-role; and feel ‘*safe enough to embrace being uncomfortable*’ [31](p340)]. In this study students who had a positive experience in the doctor-role often noted the encouraged to do this by their tutor; the supportive atmosphere afforded by small group sizes; tutor acknowledgement that the simulation was not real clinical practice; and clarifying expectations and exploring concerns. This in line with advice about how best to create a psychologically safe learning environment and that acknowledging and negotiating the ‘reality gap’ supports psychological safety [31].

### Transformative learning and the powerful role of discomfort

Student thinking shifted from shorter-term, performance outcomes (e.g., expectations of an OSCE exam) to more professional foci of patient care and implications of their actions. This change of perspective signals transformative learning where the student mindset changes and can expand to become ‘*more inclusive, discriminating, open, reflective, and emotionally able to change*’ [32](p.58)]. Simulation can lead to a profound shift in outlook and different ways of understanding or being; for example learning the importance of speaking up in difficult situations [5]. Although these processes may occur irrespective of role played, this study has shown that encouraging students to adopt the doctor role has potential to instigate transformative learning.

Discomfort is recognised as an intrinsic part of transformative learning [33] and can signal that student learning is being centred around the zone of proximal development [34]: many students recognised the value of being pushed out of their comfort zone and the educational benefit of this. This pivotal role of discomfort in simulation has been noted in other studies, with discomfort being accompanied by progress, learning from mistakes, learning new concepts and considering new identities [5,35].

Despite concerns that adopting the doctor role might be too challenging for junior students, the majority of students reported the ‘doctor’ role; and no students reported undue anxiety during the simulation. There have been concerns that a negative experience might lead to loss of confidence [12] however King et al [19] point out the risk of a negative experience in simulation where an appropriate de-brief can occur is often preferable to a negative clinical situation in real-life [19]. Our study demonstrated that once able to address their concerns and enabled to navigate appropriate psychological fidelity, junior students respond positively to being encouraged to take on a doctor-role

### A trigger for professional conversations including managing uncertainty

The debrief component of simulation is crucial in how learners interpreted and contextualise their experience [5]. It is a powerful vehicle for critical discourse, where learners gain feedback, and reflective discussion can facilitate deep learning and development of new perspectives [34,36]. In this study, important professionalism conversations were seen taking place during the debrief. Whilst these may have taken place independent of the role that the student played, engagement with the new role prompted productive conversations about working with uncertainty in clinical practice. Tutors were seen to challenge expectations of certainty, and unrealistic expectations that the ‘doctor will know everything’. There has been criticism of simulation that it can prioritise simpler ‘typical’ presentations over more authentic, complex cases in the pursuit of specific learning outcomes [37,38]; however primary care simulations have already demonstrated that they are excellent vehicles to help address uncertainty whilst navigating complex situations [39]. There have been calls to address certainty more explicitly within medical training and to challenge unrealistic expectations of certainty [40,41] and this study has identified a way to do this.

There has been concern that, in the early clinical years, clinical experience is often not contextualised through appropriate discussion [3] and prompting students to become more doctor-centred and with reduced empathy levels [42]. Negotiation of the doctor-role was seen to open up reflective conversations between students and faculty about their developing role -a positive move as facilitating space for these conversations may help develop empathy; safeguard against cynicism and burnout [43]; and promote resilience [44].

There have been calls to give more focus to professional identity formation [15], particularly for those who might feel that there are stereotypes within medicine with which they don’t fit [45]. Brown [46] argues that discussion with faculty might help to challenge existing structural prejudices and inequalities and this is an area in which further research should be done. The exercise of ‘stepping into the role of a doctor’, and conversations that ensue, thus has much potential to open up important conversations that promote more inclusive development of professional identity.

Limitations:

Methods in this study enabled a holistic insight into the phenomenon from multiple avenues [47], however, as in most case studies, the primary instrument of data collection is the researcher, which is dependent on an individual’s skills, perspectives and biases [23]; in this case primarily a faculty member with a role in organising the sessions. Two authors (NJ, RH) are insider researchers who have had input into design of these simulations.

This study considers the contrast between learners role-playing as either doctor or student, however these roles often overlap in the simulation context making it challenging to delineate the value of role-playing a doctor from the overall simulation experience.

## Conclusion

This study demonstrates the educational value of allowing junior medical students to ‘step into the role of a doctor’. The value of adopting the doctor-role pivots around a novel experience of clinical responsibility and agency; that exposes learners to a high-fidelity experience of managing uncertainty in a clinical context. Encouragement from tutors and a psychologically safe environment are essential to facilitate student engagement with the role. Exploration and negotiation of student concerns triggered valuable reflective conversations.
